# Expression and function of inducible co-stimulator in patients with systemic lupus erythematosus: possible involvement in excessive interferon-γ and anti-double-stranded DNA antibody production

**DOI:** 10.1186/ar1928

**Published:** 2006-03-22

**Authors:** Manabu Kawamoto, Masayoshi Harigai, Masako Hara, Yasushi Kawaguchi, Katsunari Tezuka, Michi Tanaka, Tomoko Sugiura, Yasuhiro Katsumata, Chikako Fukasawa, Hisae Ichida, Satomi Higami, Naoyuki Kamatani

**Affiliations:** 1 Institute of Rheumatology, Tokyo Women's Medical University, Tokyo, Japan; 2 Clinical Research Center, Tokyo Medical and Dental University, Tokyo, Japan; 3 Central Pharmaceutical Research Institute, Japan Tobacco, Inc., Osaka, Japan

## Abstract

Inducible co-stimulator (ICOS) is the third member of the CD28/cytotoxic T-lymphocyte associated antigen-4 family and is involved in the proliferation and activation of T cells. A detailed functional analysis of ICOS on peripheral blood T cells from patients with systemic lupus erythematosus (SLE) has not yet been reported. In the present study we developed a fully human anti-human ICOS mAb (JTA009) with high avidity and investigated the immunopathological roles of ICOS in SLE. JTA009 exhibited higher avidity for ICOS than a previously reported mAb, namely SA12. Using JTA009, ICOS was detected in a substantial proportion of unstimulated peripheral blood T cells from both normal control individuals and patients with SLE. In CD4^+^CD45RO^+ ^T cells from peripheral blood, the percentage of ICOS^+ ^cells and mean fluorescence intensity with JTA009 were significantly higher in active SLE than in inactive SLE or in normal control individuals. JTA009 co-stimulated peripheral blood T cells in the presence of suboptimal concentrations of anti-CD3 mAb. Median values of [^3^H]thymidine incorporation were higher in SLE T cells with ICOS co-stimulation than in normal T cells, and the difference between inactive SLE patients and normal control individuals achieved statistical significance. ICOS co-stimulation significantly increased the production of IFN-γ, IL-4 and IL-10 in both SLE and normal T cells. IFN-γ in the culture supernatants of both active and inactive SLE T cells with ICOS co-stimulation was significantly higher than in normal control T cells. Finally, SLE T cells with ICOS co-stimulation selectively and significantly enhanced the production of IgG anti-double-stranded DNA antibodies by autologous B cells. These findings suggest that ICOS is involved in abnormal T cell activation in SLE, and that blockade of the interaction between ICOS and its receptor may have therapeutic value in the treatment of this intractable disease.

## Introduction

Systemic lupus erythematosus (SLE), a prototype autoimmune disease, is characterized by activation of lymphocytes and the presence of various types of autoantibodies in peripheral blood. These autoantibodies are considered to form immune complexes with their corresponding autoantigens and to mediate tissue and organ damage [1]. Recent investigations suggest that collaboration between autoantibody-producing B cells and antigen-specific T-helper (Th) cells is important to the production of these pathogenic autoantibodies [2].

The fate of T cells, after they encounter specific antigens, is modulated by co-stimulatory signals, which are required for both lymphocyte activation and the development of adaptive immunity (for review [3-6]). In general, activation of T cells requires two signals: one from a T cell receptor and the other from co-stimulatory molecules such as CD28 and tumour necrosis factor family members [3,7]. The inducible co-stimulator (ICOS; also known as AILIM [activation-inducible lymphocyte immunomediatory molecule]) was identified in 1999 as a membrane glycoprotein that is expressed on the surface of activated T cells and that shares several structural and functional similarities with CD28 [8-10]. Like CD28, ICOS has potent co-stimulatory effects on proliferation of T cells and production of cytokines [8-12]. ICOS is also important for germinal centre formation, clonal expansion of T cells, antibody production, and class switching in response to various antigens [13,14]. CD28 and cytotoxic T lymphocyte associated antigen 4 use the MYPPPY motif in their extracellular domains to bind to their ligands, namely B7.1 and B7.2. ICOS does not possess this motif, and so B7.1 and B7.2 are not among its ligands [9]. Subsequently, it was shown that a B7-like molecule, termed B7-related protein-1 (B7RP-1) (also referred to as B7-H2, GL50 and LICOS), binds to ICOS [9,15-21]. B7RP-1 shares 20% identity with B7.1/B7.2 [9] and is constitutively expressed on B cells and monocytes [13].

Accumulating evidence indicates that ICOS is involved in the immunopathogenesis of animal models of various autoimmune disorders, including SLE, rheumatoid arthritis, multiple sclerosis and asthma [21-28]. These data prompted us to investigate the possible role of ICOS in human SLE and its importance as a therapeutic target. We found that ICOS was over-expressed in peripheral blood CD4^+ ^T cells from patients with active SLE and that ICOS contributed not only to the enhanced proliferation but also to the increased production of IFN-γ in peripheral blood T cells from patients with SLE. ICOS also augmented the ability of peripheral blood T cells from patients with SLE to support the production of IgG anti-double stranded (ds)DNA antibody by autologous peripheral blood B cells. Thus, we examined the expression and function of ICOS in peripheral blood T cells from patients with SLE. Our data suggest that ICOS plays an important role in the immunopathogenesis of SLE and support the possibility that blockade of the interaction between ICOS and B7RP-1 may have therapeutic value in treating this intractable autoimmune disorder.

## Materials and methods

### Patients

Twenty-two patients with active SLE (21 females and one male), 17 patients with inactive SLE (16 females and one male) and 24 normal control individuals (22 females and two males) were included in the study. All SLE patients fulfilled the SLE classification criteria proposed by the American College of Rheumatology [29]. Disease activity in the SLE patients was evaluated using the Systemic Lupus Erythematosus Disease Activity Index (SLEDAI) [30]. SLEDAI scores for the patients with active SLE ranged from 6 to 22 (mean ± standard deviation [SD] 10.0 ± 6.2; median 10), whereas the scores for the patients with inactive SLE ranged from 0 to 2 (mean ± SD 0.9 ± 1.0; median 0). Sixteen of the 22 patients with active SLE were examined before administration of corticosteroids and immunosuppressants. Treatments for the remaining six patients with active SLE were as follows: low-dose prednisolone (≤ 15 mg/day, median 9.5 mg/day; *n *= 4); 30 mg/day prednisolone (*n *= 1); and 100 mg/day prednisolone and 250 mg/day cyclosporine A (*n *= 1). Sixteen of the 17 patients with inactive SLE were treated with low-dose prednisolone (median 10 mg/day); the remaining patients had been followed up without medication.

Peripheral blood samples were obtained with the informed consent of all participating individuals. The Helsinki Declaration was adhered to throughout the study.

### Generation of fully human anti-ICOS monoclonal antibody (JTA009)

The generation and characterization of the Xeno-Mouse-G2 strains, engineered to produce fully human IgG_2 _antibodies, were described by Mendez and coworkers [31]. Xeno-Mouse-G2 mice (aged 8–10 weeks) were immunized with a footpad injection of the membrane fraction isolated from human ICOS expressing CHO-K1 cells [32] in complete Freund's adjuvant. Mice were boosted with the same amount of the fraction three to four times before fusion. Popliteal lymph node and spleen cells were fused with the murine myeloma cell line P3X63Ag8.653 (CRL-1580; American Type Culture Collection, Manassas, VA, USA) using PEG1500. Hybridomas were screened for their ability to bind to human ICOS expressed on CHO-K1 or HPB-ALL cells [32]. One of the mAbs, JTA009, exhibited high avidity for human ICOS and was used in the following experiments. The characteristics of JTA009 are described below in the Results section. JMAb23, a class-matched control mAb for JTA009, was generated against keyhole limpet hemocyanin (KLH) in the same manner. All experiments were conducted following institutional guidelines for the ethical treatment of animals.

### Other antibodies

The anti-human ICOS mAb SA12 was generated and characterized as described previously [32]. Anti-CD3 mAb (clone UCHT1) and anti-CD28 mAb (clone 28.2) were obtained from Beckman Coulter Inc. (Fullerton, CA, USA). Anti-B7RP-1 mAb was obtained from R&D Systems (Minneapolis, MN, USA). Fluorescein isothiocyanate (FITC)-conjugated anti-CD3 mAb was purchased from DAKO Japan (Tokyo, Japan). Phycoerythrin (PE)-conjugated anti-CD45RO mAb and PE-conjugated control IgG were obtained from Nichirei (Tokyo, Japan). PE-conjugated anti-CD25 mAb was obtained from eBioscience (San Diego, CA, USA). PE-conjugated anti-CD69 mAb and peridinin chlorophyll protein (PerCP)-conjugated mAbs to human CD3, CD4 and CD8 were purchased from BD Biosciences (San Jose, CA, USA). The F(ab')_2 _fraction of goat anti-human IgG antibody was obtained from Biosource International Inc. (Camarillo, CA, USA). Peroxidase-conjugated anti-human IgG was obtained from MBL (Nagoya, Japan).

### Cell preparations

Peripheral blood lymphocytes (PBLs) were separated by centrifugation of heparinized blood over a Ficoll-Conray gradient. B cells were isolated by positive selection from PBLs using anti-CD19 MicroBeads (Miltenyi Biotech, Auburn, CA, USA), in accordance with the manufacturer's instructions. T cells were selected from CD19-depleted PBLs using the Pan T cell Isolation Kit (Miltenyi Biotech) and anti-CD14 MicroBeads (Miltenyi Biotech). The purities of B cells and T cells were in excess of 97% and 95%, respectively, using flow cytometry.

### Immunoprecipitation and Western blotting

Peripheral blood T cells from normal control individuals were stimulated with anti-CD3 mAb (0.1 μg/ml) + anti-CD28 mAb (2 μg/ml) for 72 hours. The surface of these cells was biotinylated using the ECL Protein Biotinylation Module (Amersham Bioscience Corp., Piscataway, NJ, USA) and lysates were prepared with lysis buffer containing 25 mmol/l Tris-HCl (at pH 7.5), 250 mmol/l NaCl, 5 mmol/l EDTA, 1% NP-40, protease inhibitor cocktail (Roche Diagnostics GmbH, Mannheim, Germany) and 1 mmol/l phenylmethanesulfonyl fluoride. JTA009 or JMAb23 were conjugated with Protein G-agarose (Pierce Biotechnology Inc., Rockford, IL, USA) and incubated with the cell lysate at 4°C overnight. After washing three times with lysis buffer, the mAb-conjugated Protein G-agarose was boiled for two minutes and the bound antigens were separated using 12.5% SDS-PAGE gel and transferred to nitrocellulose membrane (Bio-Rad Laboratories, Hercules, CA, USA). Transferred protein was visualized using streptavidin-peroxidase (Amersham Bioscience Corp.) and SuperSignal West Pico Chemiluminescent Substrate (Pierce Biotechnology Inc.).

### Flow cytometry

Multicolour analysis was performed using flow cytometry. Cells were washed three times in ice cold FCM buffer (phosphate-buffered saline [PBS] containing 0.1% bovine serum albumin and 0.1% sodium azide) and incubated on ice for five minutes with 10 μg purified human immunoglobulin (Cappel, ICN, Aurora, OH, USA) and/or 10 μg purified mouse IgG (Chemicon, Temecula, CA, USA) to block nonspecific IgG binding. Cells were then incubated at 4°C with saturating amounts of the fluorochrome (for instance, FITC, PE, or PerCP) or biotin conjugated mAbs for 30 minutes. Cells were washed twice in ice cold FCM buffer and incubated at 4°C with streptavidin-FITC (DAKO Japan) for 30 minutes. After incubation, cells were washed three times in ice cold FCM buffer and fixed in PBS containing 1% paraformaldehyde. The expression of cell surface markers was evaluated using an EPICS^® ^ALTRA (Beckman Coulter Inc.) cell sorter and EXPO32™ analysis software (Beckman Coulter Inc.).

### Stimulation of T cells

Peripheral blood T cells were stimulated either with anti-CD3 mAb (0.1 μg/ml) plus anti-CD28 mAb (2 μg/ml; CD28 costimulation), or with anti-CD3 mAb (0.1 μg/ml) plus JTA009 (8 μg/ml; ICOS costimulation). Anti-CD3 mAb and JTA009 were bound to flat-bottomed 96-well microtitre plates (IWAKI, Tokyo, Japan) by incubating overnight at 4°C. Preliminary experiments showed that anti-CD3 mAb alone at 0.1 μg/ml induced modest proliferation of peripheral blood T cells under the conditions described above (data not shown). In some experiments, T cells were stimulated with anti-CD3 mAb plus anti-ICOS mAb or anti-CD3 plus anti-CD28 mAb in the presence of various concentration of B7RP-1-Fc (R&D Systems; 165-B7). To determine proliferative response, T cells (2 × 10^5 ^cells/well) were cultured for 72 hours with or without stimuli and pulsed with [^3^H]thymidine (1 μCi/well; Amersham Bioscience Corp.) for the last 8 hours. The uptake of [^3^H]thymidine was measured using Matrix96 (Packard Instrument Company, Meridian, CT, USA). To determine cytokine production, T cells (2 × 10^5 ^cells/well) were cultured with or without stimuli for 72 hours and culture supernatants were collected.

### T/B cell co-culture

T cells and B cells, purified from the peripheral blood of patients with active SLE with high serum levels of anti-dsDNA antibody, were reconstituted at a 1:1 ratio (1 × 10^5 ^T cells and B cells/well), and were cultured in the presence of various stimuli for seven days. Culture supernatants were collected and stored at -80°C until assayed for anti-dsDNA antibody and total IgG.

### ELISA for cytokines, IgG anti-dsDNA antibody, total IgG and anti-tetanus antibody

IL-2, IL-4, IL-10 and IFN-γ production in the culture supernatants was measured using ELISA kits, in accordance with the manufacturer's protocol (IL-2 from R&D Systems, IL-4 and IL-10 from Biosource International Inc., and IFN-γ from Amersham Bioscience Corp.). The sensitivities of these ELISA kits were 1.60 pg/ml, 0.39 pg/ml, 0.78 pg/ml and 0.63 pg/ml for IL-2, IL-4, IL-10 and IFN-γ, respectively. IgG anti-dsDNA antibody and total IgG in culture supernatants were determined as described previously [33]. Anti-tetanus antibody was measured using ELISA kits from Virion/Serion (Würzburg, Germany), in accordance with the manufacturer's protocol.

### ELISA for anti-ICOS mAbs

To compare the sensitivities of JTA009 and SA12, ELISA for anti-ICOS mAbs was performed. Both antibodies and JMAb23 were biotinylated using FluoReporter^® ^Mini-biotin-XX Protein Labeling Kit (Invitrogen Japan K.K., Tokyo, Japan), in accordance with the manufacturer's instructions. Biotinylation was confirmed by coating ELISA plates with serial dilutions of the biotinylated mAbs and detecting them with streptavidin-HRP (DAKO) and TMB+ substrate chromogen (DAKO). Both antibodies were biotinylated at the same level. Then, various amounts of ICOS-Fc (R&D Systems) were coated on the ELISA plate at 4°C overnight. After blocking the wells with PBS containing 0.01% Tween-20 (PBS-T) plus 1% casein, 50 μL of 0.3 μg/ml biotinylated anti-ICOS mAb (JTA-009 or SA12) or isotype-matched control antibody was added to the wells and incubated at room temperature for 1 hour. After washing away any unbound biotinylated antibody with PBS-T, 50 μl of 1/1000 diluted streptavidin-horseradish peroxidase was added. After incubation at room temperature for 1 hour, the plate was washed with PBS-T to remove unbound conjugate. TMB+ substrate chromogen was added to the wells. After stopping the colorization with 0.1 mol/l H_2_SO_4 _(Wako), the optical density was measured at 450 nm using a spectrophotometer.

### Statistical analysis

Values are expressed as mean ± SD, unless otherwise stated. The differences between groups were evaluated using Mann-Whitney *U *test. Paired samples were analyzed using Wilcoxon's rank sum test. *P *< 0.05 was considered statistically significant.

## Results

### Characterization of JTA009, a newly developed human anti-ICOS mAb

**Figure 1:**
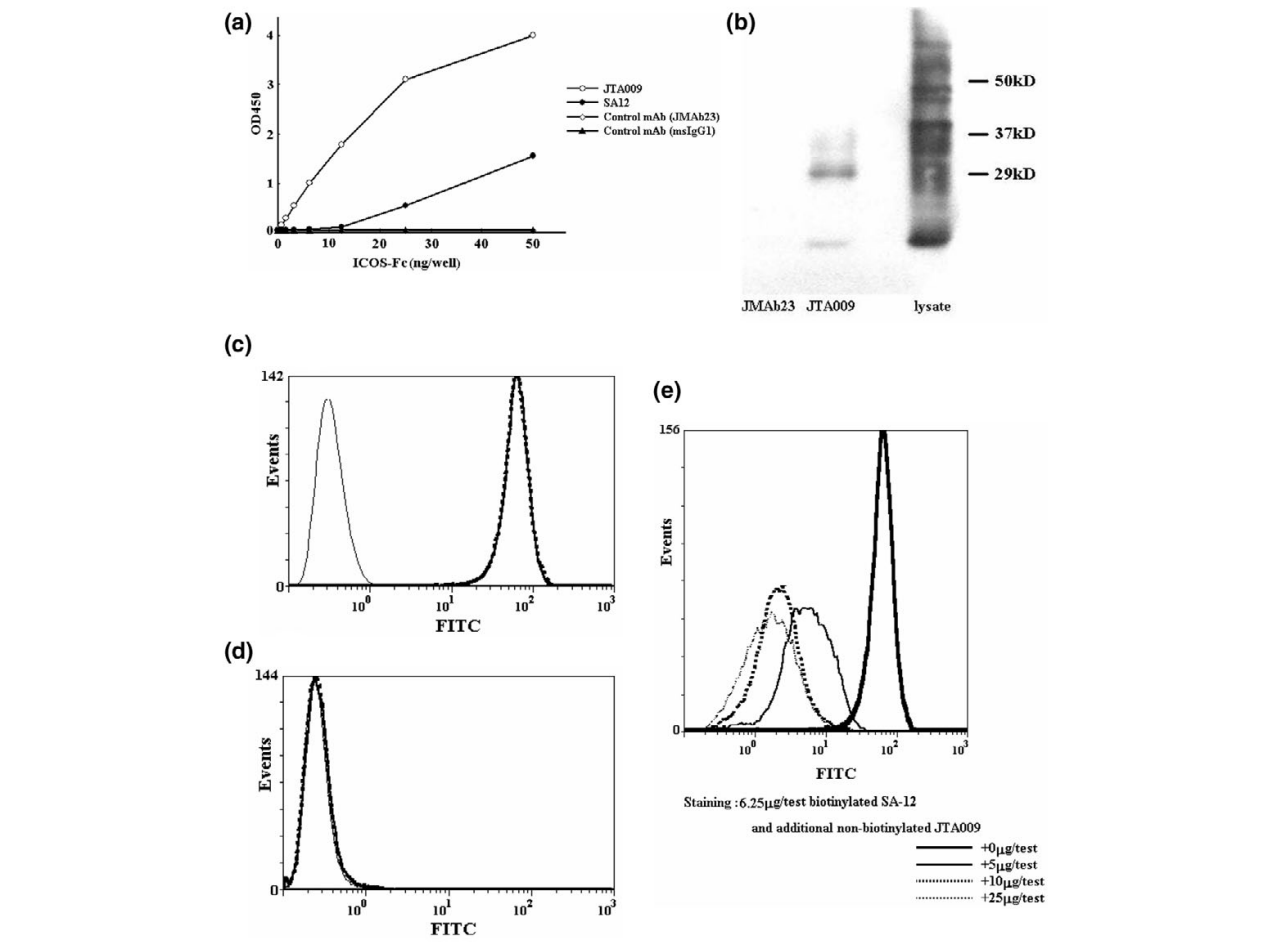
Characterization of JTA009, a novel anti-human ICOS mAb. JTA009, a fully human anti-human ICOS mAb, has greater avidity than SA12. **(a) **Avidity of anti-human ICOS antibodies was evaluated by direct ELISA using ICOS-Fc (as described in Materials and method). JTA009 (open circles) exhibited stronger binding to ICOS-Fc than did SA12 (closed circle), a previously reported anti-human ICOS mAb. **(b) **Peripheral blood T cells from normal control individuals were stimulated with anti-CD3 mAb (0.1 μg/ml) plus anti-CD28 mAb (2 μg/ml) for 72 hours. These cells were biotinylated and cell lysates were prepared. ICOS molecules in these lysates were immunoprecipitated, separated on SDS-PAGE gel, transferred to nitrocellulose membrane, and visualized using streptavidin-peroxidase and chemiluminescent substrate. A single band about 29 kDa was immunoprecipitated with JTA009 but not with JMAb23, the control antibody. The thin lower band corresponded to the position of the front dye of the gel. Human ICOS expressing **(c) **CHO-K1 and **(d) **its parental cell line CHO-K1 were stained with biotinylated JTA009 (thick line), biotinylated SA12 (broken line), or biotinylated JMAb23 (human IgG_2_; thin line) and streptavidin-FITC, and then analyzed using flow cytometry. **(e) **Human ICOS expressing CHO-K1 cells were stained biotinylated SA12 (6.25 μg/test) and streptavidin-FITC in the presence of various amounts of nonbiotinylated JTA009 (thick line: 0 μg/test; thin line: 5 μg/test; thick broken line: 10 μg/test; thin broken line: 25 μg/test). JTA009 dose dependently decreased the binding of SA12 to the ICOS expressing CHO-K1 cells. FITC, fluorescein isothiocyanate; ICOS, inducible co-stimulator; mAb, monoclonal antibody.

We initially conducted experiments to characterize JTA009, the newly developed human anti-human ICOS mAb (Figure 1). Direct ELISA using a recombinant ICOS-Fc coated plate clearly showed that JTA009 had greater avidity for the ICOS molecule than did the previously reported anti-human ICOS mAb SA12 (Figure 1a). We confirmed the specificity of JTA009 by immunoprecipitation. JTA009 immunoprecipitated a 29 kDa band (corresponding to the molecular weight of human ICOS) on activated peripheral blood T cells, but the control antibody JMAb23 did not (Figure 1b).

We then compared both anti-human ICOS mAbs using flow cytometry. Both anti-ICOS mAbs bound to human ICOS expressing CHO-K1 (CCL61) cells (Figure 1c) but not to control CHO-K1 cells (Figure 1d), indicating the specificity of these two mAbs. Furthermore, binding of biotinylated SA12 to ICOS expressing CHO-K1 cells was dose-dependently replaced by nonbiotinylated JTA009 (Figure 1e). These data strongly indicated that JTA009 was specific to human ICOS and had greater avidity than SA12.

We also compared the binding profiles of SA12 and JTA009 to peripheral blood T cells from 11 normal control individuals. Percentages of cells positive for JTA009 were 29.2 ± 22.1% and 11.6 ± 11.2% (mean ± SD) for peripheral blood CD4^+ ^and CD8^+ ^T cells, respectively. These values were significantly higher than those of SA12, which were 3.8 ± 2.4% for CD4^+ ^T cells (*P *= 0.0033) and 1.6 ± 1.0% for CD8^+ ^T cells (*P *= 0.0033; Table 1). We also performed multicolor staining and analyzed the relationship between ICOS and CD45RO in peripheral blood T cells. When JTA009 was used, percentages of ICOS^+ ^cells on CD4^+^CD45RO^+ ^and CD8^+^CD45RO^+ ^normal peripheral blood T cells were 37.3 ± 25.8% and 17.1 ± 15.2%, respectively, which were significantly higher than the corresponding percentages using SA12 (*P *= 0.0033; Table 1). We compared mean fluorescence intensity (MFI) for ICOS expression in CD45RO^+ ^memory T cells and CD45^- ^naïve T cells using JTA009. MFI for ICOS expression in CD4^+^CD45RO^+ ^T cells and CD8^+^CD45RO^+ ^T cells was significantly higher than that in CD4^+^CD45RO^- ^T cells and CD8^+^CD45RO^- ^T cells, respectively (CD4^+^CD45RO^+^: 0.93 ± 0.38; CD4^+^CD45RO^-^: 0.42 ± 0.19; CD8^+^CD45RO^+^: 0.42 ± 0.25; CD8^+^CD45RO^-^: 0.19 ± 0.16; *P *= 0.0033 for CD4^+ ^T cells and *P *= 0.0022 for CD8^+ ^T cells). Thus, compared with SA12, JTA009 possesses a stronger binding profile and is more sensitive in detecting the expression of ICOS on human T cells.

### Augmented expression of ICOS on peripheral blood CD4^+ ^T cells from patients with active SLE

**Figure 2:**
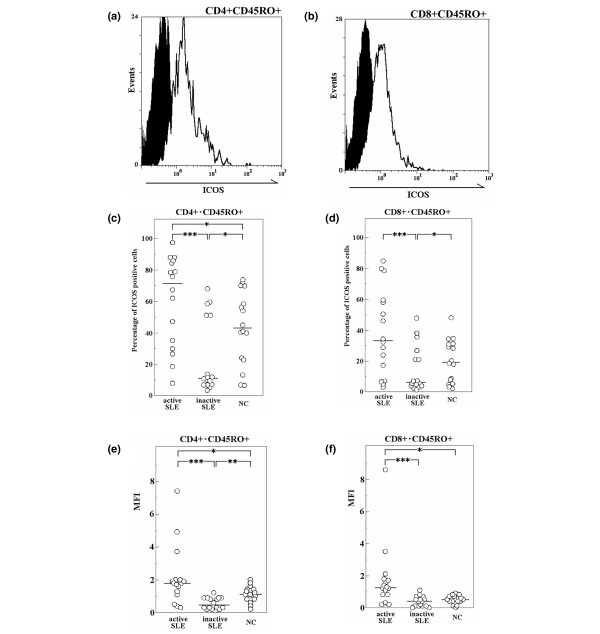
Expression of ICOS on peripheral blood T cells from SLE patients and normal control individuals. Peripheral blood T cells were analyzed using three-colour staining (anti-CD4-PerCP or anti-CD8-PerCP, anti-CD45RO-PE, and biotinylated JTA009 plus streptavidin-FITC) and flow cytometry for ICOS expression. Representative patterns of ICOS expression on **(a) **CD4^+^CD45RO^+ ^and **(b) **CD8^+^CD45RO^+ ^peripheral blood T cells from a patients with active SLE are shown. The background histograms (shown in black) were obtained by staining with anti-CD4-PerCP or anti-CD8-PerCP, anti-CD45RO-PE, and biotinylated JMAb23 (control mAb) plus streptavidin-FITC. **(c-f) **Peripheral blood T cells from patients with active SLE (*n *= 16), patients with inactive SLE (*n *= 16) and normal control individuals (*n *= 16) were analyzed using three-color staining and flow cytometry for ICOS expression. Percentages of ICOS^+ ^cells (panels c and d) and MFIs of ICOS^+ ^cells (panels e and f) are shown. CD4^+^CD45RO^+ ^(panels c and e) and CD8^+^CD45RO^+ ^(panels d and f) peripheral blood T cells were analyzed. Bars indicate median values of each group. Percentages (medians) of CD4^+^CD45RO^+ ^ICOS^+ ^cells and CD8^+^CD45RO^+^ICOS^+ ^cells, respectively, were as follows: active SLE, 71.3% and 33.2%; inactive SLE, 11.1% and 6.2%; and normal control individuals, 42.8% and 19.2%. The MFI (medians) of CD4^+^CD45RO^+ ^ICOS^+ ^cells and CD8^+^CD45RO^+^ICOS^+ ^cells, respectively, were as follows: active SLE, 1.80 and 1.25; inactive SLE, 0.45 and 0.40; and normal control individuals, 1.10 and 0.50. *P < 0.05, **P < 0.01, and ***P < 0.005, by Mann-Whitney *U*-test. FITC, fluorescein isothiocyanate; ICOS, inducible co-stimulator; mAb, monoclonal antibody; MFI, mean fluorescence intensity; NC, normal control; PE, phycoerythrin; PerCP, peridinin chlorophyll protein; SLE, systemic lupus erythematosus.

Peripheral blood T cells from SLE patients and normal control individuals were analyzed for expression of ICOS using three-color staining and flow cytometry. Because ICOS was predominantly expressed on CD45RO^+ ^T cells in normal control individuals as well as in patients with SLE (Table 1, Figure 2 and data not shown), we gated on either CD4^+^CD45RO^+ ^or CD8^+^CD45RO^+ ^T cells and analyzed the expression of ICOS on these subsets (Figure 2a–f). We determined the cutoff points for positive staining so that the percentage of positive cells with control antibody JMAb23 was less than 1%. The percentage of CD4^+^CD45RO^+ ^T cells expressing ICOS in active SLE was significantly greater than the percentages in inactive SLE and normal control individuals. Interestingly, percentages of both CD4^+^CD45RO^+ ^and CD8^+^CD45RO^+ ^T cells expressing ICOS in inactive SLE were significantly lower than those in active SLE and normal control (Figure 2c,d). The MFIs of ICOS on both CD4^+^CD45RO^+ ^and CD8^+^CD45RO^+ ^T cells from patients with active SLE were significantly higher than those in inactive SLE patients and normal control individuals (Figure 2e,f). There was no significant correlation between SLEDAI score and expression of ICOS in these patients with SLE. We examined expression of ICOS in three patients with active SLE before and after treatment with high-dose prednisolone. In these three cases, percentages of ICOS on both CD4^+^CD45RO^+ ^and CD8^+^CD45RO^+ ^T cells drastically decreased (CD4^+^CD45RO^+^: 71.0 ± 11.7% before treatment versus 13.4 ± 5.0% after treatment; CD8^+^CD45RO^+^: 45.2 ± 12.9% before treatment versus 10.3 ± 6.8% after treatment).

### Proliferative response of peripheral blood T cells to ICOS co-stimulation

**Figure 3:**
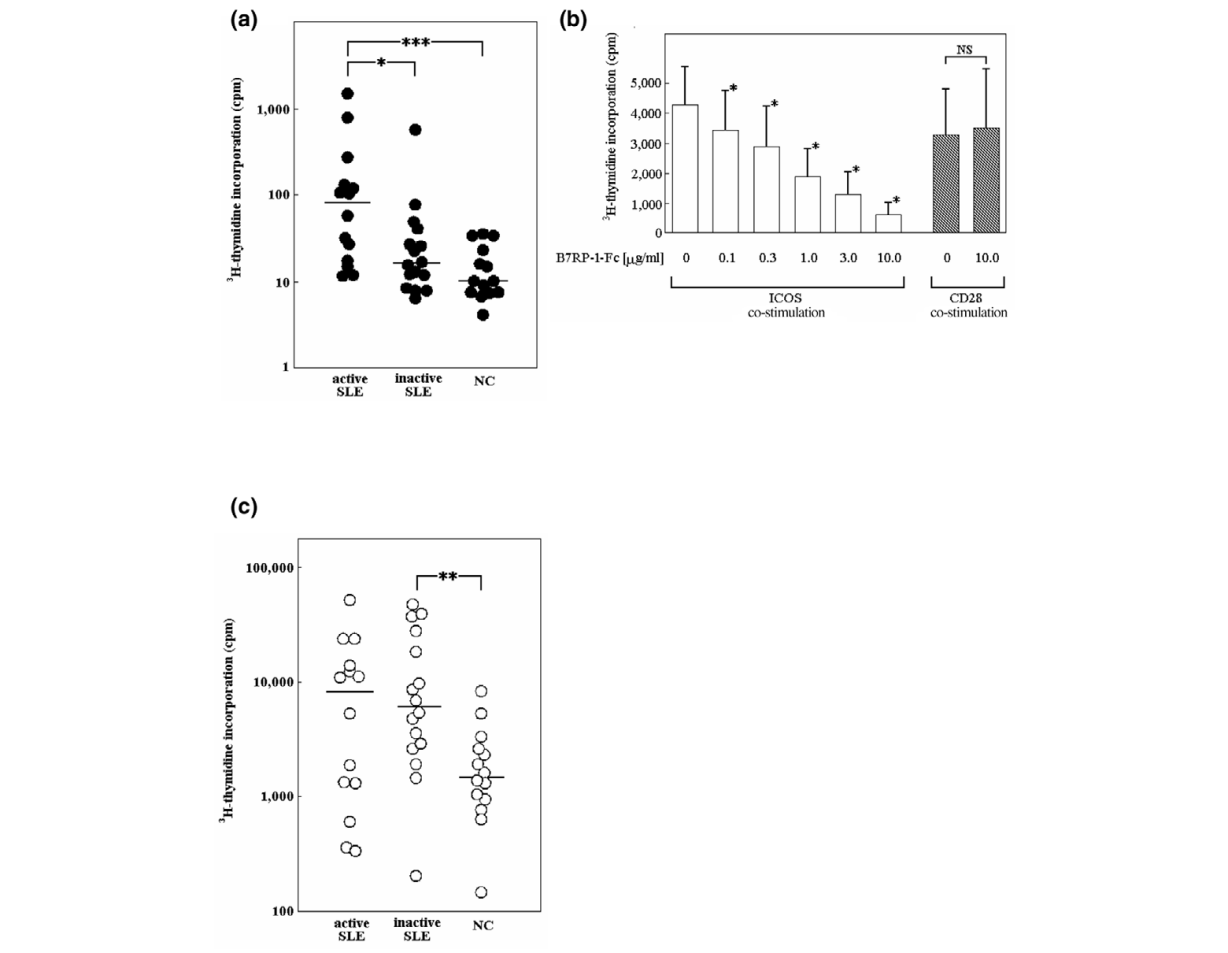
Proliferative response of peripheral blood T cells to ICOS co-stimulation. Peripheral blood T cells isolated from patients with active SLE (*n *= 14), patients with inactive SLE (*n *= 16), and normal control individuals (*n *= 14) were cultured for 72 hours with or without stimulation and pulsed with [^3^H]thymidine during the last 8 hours. **(a) **[^3^H]thymidine incorporation without stimulation. The median value of each group was as follows: active SLE, 78.9 counts/min; inactive SLE, 15.9 counts/min; and normal control individuals, 9.9 counts/min. **(b) **Inhibition of ICOS co-stimulation by B7RP-1. Peripheral blood T cells from normal control individuals were stimulated with either anti-CD3 mAb plus JTA009 or anti-CD3 mAb plus anti-CD28 mAb in the presence of various concentration of B7RP-1-Fc. Proliferation of peripheral blood T cells with ICOS co-stimulation, but not that with CD28 co-stimulation, was dose dependently inhibited by the addition of B7RP-1-Fc to cell culture medium. **(c) **[^3^H]thymidine incorporation with ICOS co-stimulation. The median values in each group for ICOS co-stimulation were as follows: active SLE, 8063 counts/min; inactive SLE, 6050 counts/min; and normal control individuals, 1481 counts/min. Bars indicate median values in each group. *P < 0.05, **P < 0.01, ***P < 0.005 by Mann-Whitney *U*-test. B7RP, B7-related protein; ICOS, inducible co-stimulator; mAb, monoclonal antibody; NC, normal control; SLE, systemic lupus erythematosus.

We then investigated the effects of ICOS co-stimulation on the proliferation of peripheral blood T cells. The [^3^H]thymidine incorporation of unstimulated peripheral blood T cells from active SLE patients was significantly greater than that for patients with inactive SLE (*P *< 0.05) and normal control individuals(*P *< 0.005), indicating that peripheral blood T cells from active SLE patients were already activated *in vivo *(Figure 3a). Peripheral blood T cells were stimulated with suboptimal concentrations of anti-CD3 mAb (0.1 μg/ml) and optimal concentrations of anti-ICOS mAb or anti-CD28 mAb, as described above under Materials and method. Anti-CD3 mAb alone at this concentration induced modest proliferation of peripheral blood T cells. CD28 co-stimulation was used as a positive control. With the above experimental conditions, ICOS co-stimulation as well as CD28 co-stimulation significantly increased [^3^H]thymidine incorporation for normal peripheral blood T cells (*n *= 14; without stimulation: 15 ± 11 counts/minute; ICOS co-stimulation: 2244 ± 2160 counts/minute; CD28 co-stimulation: 3101 ± 1900 counts/minute; *P *< 0.001 for both co-stimulations versus without stimulation). Proliferation of peripheral blood T cells with ICOS co-stimulation in normal control individuals, but not that with CD28 co-stimulation, was dose-dependently inhibited by the addition of B7RP-1-Fc, indicating the involvement of ICOS-B7RP-1 interaction in anti-CD3 mAb plus JTA009 stimulation (Figure 3b). ICOS co-stimulation significantly increased the [^3^H]thymidine incorporation of peripheral blood T cells in all three groups (active SLE: *P *= 0.0012; inactive SLE: *P *= 0.0004; normal control individuals: *P *= 0.001). The [^3^H]thymidine incorporation of peripheral blood T cells from inactive SLE patients after ICOS co-stimulation was significantly higher than that for normal control individuals (*P *< 0.01; Figure 3c). Although the median value of [^3^H]thymidine incorporation of peripheral blood T cells from active SLE patients after ICOS co-stimulation was higher than those for inactive SLE patients and normal control individuals, the difference did not reach statistical significance because of the presence of some patients with active SLE who responded poorly to the co-stimulation (Figure 3c).

Because [^3^H]thymidine incorporation of T cells with ICOS co-stimulation was IL-2 dependent [11], we measured IL-2 in the culture supernatants of the above experiments at 72 hours after ICOS co-stimulation. The mean levels of IL-2 production by peripheral blood T cells were as follows: active SLE, 5.4 ± 5.5 pg/ml (*n *= 11); inactive SLE, 6.3 ± 4.6 pg/ml (*n *= 10); and normal control individuals, 10.6 ± 10.8 pg/ml (*n *= 12). Although these mean values for patients with SLE were lower than that in normal control individuals, there was no statistical difference between the groups. These data indicate that the augmented proliferation of peripheral blood T cells from patients with inactive SLE in response to ICOS co-stimulation did not result from over-production of IL-2.

Enhanced IFN-γ production of peripheral blood T cells from SLE patients with ICOS co-stimulation.

**Figure 4:**
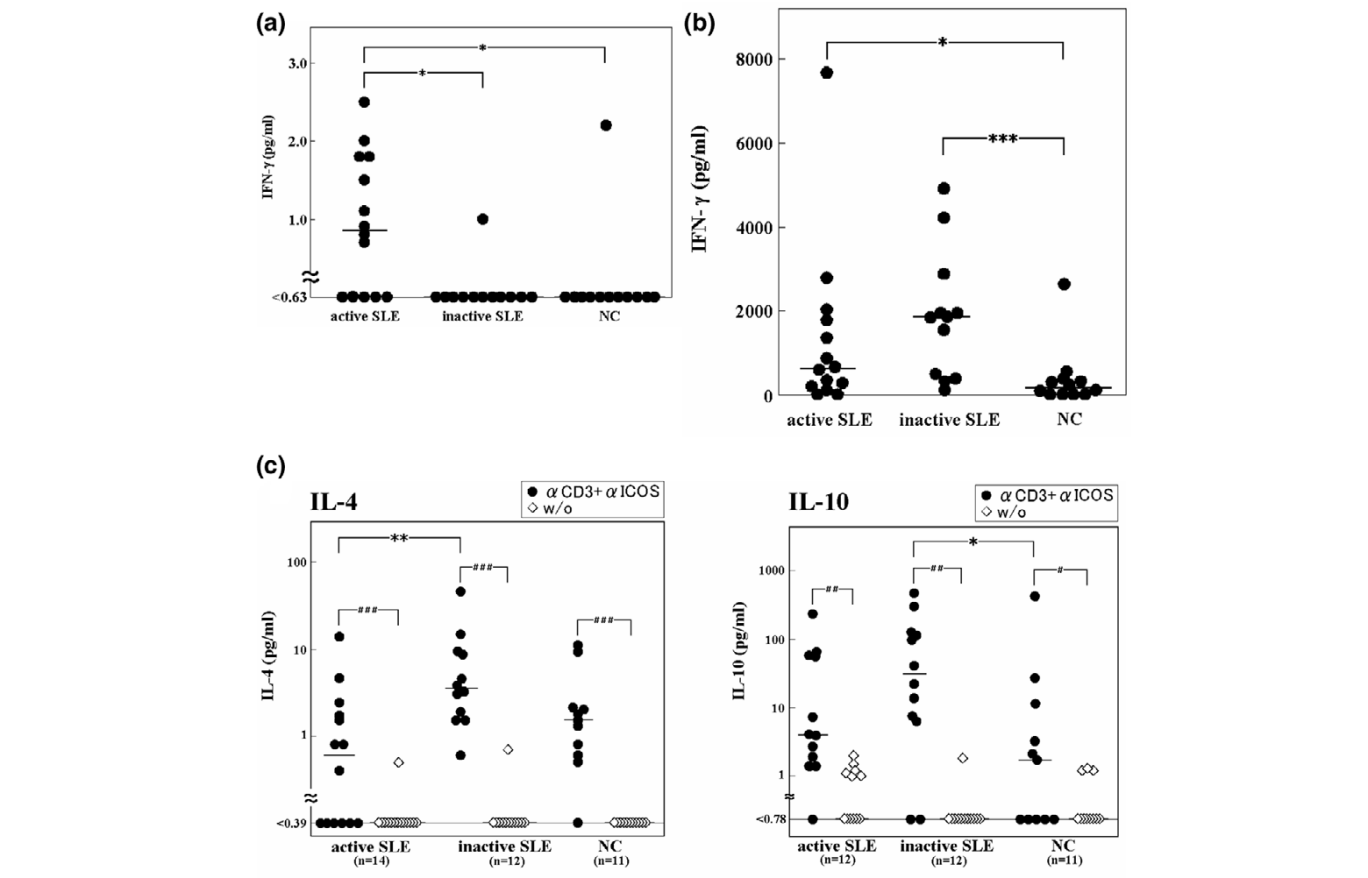
Cytokine production by peripheral blood T cells from SLE patients after ICOS co-stimulation. Peripheral blood T cells were isolated from patients with active SLE (*n *= 14), patients with inactive SLE (*n *= 12) and normal control individuals (*n *= 12) and cultured with or without ICOS co-stimulation for 72 hours; the culture supernatants were collected and the production of IFN-γ, IL-4 and IL-10 were determined by ELISA. **(a) **Production of IFN-γ without stimulation. **(b) **Production of IFN-γ with ICOS co-stimulation. **(c) **The production of IL-4 and IL-10 with or without ICOS co-stimulation. *P < 0.05, **P < 0.01, ***P < 0.005 by Mann-Whitney U-test. #*P *< 0.05, ##*P *< 0.01, ###*P *< 0.005 by Wilcoxon rank sum test. ICOS, inducible co-stimulator; NC, normal control; SLE, systemic lupus erythematosus.

Previous reports revealed immunopathological roles of IFN-γ in both human and murine lupus [34-40]. We therefore examined the effects of ICOS co-stimulation on production of IFN-γ by peripheral blood T cells. Peripheral blood T cells were cultured with or without ICOS co-stimulation for 72 hours, and the production of IFN-γ in the culture supernatants was measured using ELISA. Peripheral blood T cells from active SLE patients spontaneously produced significantly larger amounts of IFN-γ than did those from patients with inactive SLE and normal control individuals (median values: active SLE, 0.85 pg/ml; inactive SLE, <0.63 pg/ml [*P *< 0.05]; normal controls, <0.63 pg/ml [*P *< 0.05]; Figure 4a). ICOS co-stimulation of peripheral blood T cells significantly increased the production of IFN-γ in all three groups (median values: active SLE, 612.8 pg/ml [*P *< 0.001]; inactive SLE, 1843.1 pg/ml [*P *< 0.005]; normal control individuals, 174.9 pg/ml [*P *< 0.05]). Peripheral blood T cells from active and inactive SLE patients after ICOS co-stimulation produced significantly larger amounts of IFN-γ than did those from normal control individuals (*P *< 0.05 for active SLE, *P *< 0.005 for inactive SLE; Figure 4b). The enhanced production of IFN-γ in patients with SLE was also observed for CD28 co-stimulation, with a significant difference between patients with inactive SLE and normal control individuals (median values: active SLE, 370.9 pg/ml; inactive SLE, 1292.6 pg/ml; normal control individuals, 171.6 pg/ml; *P *< 0.01, patients with inactive SLE versus normal control individuals). Because ICOS has been shown to induce Th2-type cytokines, we measured IL-4 and IL-10 in the same culture supernatants [41,42]. ICOS co-stimulation of peripheral blood T cells significantly increased the production of both IL-4 and IL-10 in all three groups. Peripheral blood T cells from patients with inactive SLE after ICOS co-stimulation produced significantly larger amounts of IL-4 or IL-10 than did those from patients with active SLE or normal control individuals (*P *< 0.01 for IL-4, *P *< 0.05 for IL-10; Figure 4c)

### Effects of dexamethasone on induction of ICOS in peripheral blood T cells

Although the percentages of ICOS on both CD4^+^CD45RO^+ ^and CD8^+^CD45RO^+ ^T cells from more than half of the patients with inactive SLE were relatively low (Figure 2c,d), peripheral blood T cells from these patients with inactive SLE exhibited significantly higher proliferative response (Figure 3) and IFN-γ production (Figure 4) with ICOS co-stimulation than did cells from normal control individuals. We therefore examined expression of ICOS on peripheral blood T cells after ICOS co-stimulation in patients with inactive SLE and normal control individuals. Because JTA009, an anti-ICOS mAb, was bound to the microtitre plates during ICOS co-stimulation (as described above, under Materials and method), it did not interfere with subsequent detection of ICOS molecule on stimulated T cells. ICOS co-stimulation of peripheral blood T cells for 48 or 72 hours significantly enhanced expression of ICOS on CD3^+^CD45RO^+ ^T cells in both patients with inactive SLE and normal control individuals (patients with inactive SLE: 12.6 ± 3.9% before stimulation versus 27.5 ± 18.7% 48 hours after stimulation versus 63.5 ± 3.3 % 72 hours after stimulation; normal control individuals: 33.6 ± 28.0% before stimulation versus 53.2 ± 26.9% 48 hours after stimulation versus 67.2 ± 29.3% 72 hours after stimulation; *P *< 0.05 for both 48 and 72 hours compared with before stimulation in each group).

**Figure 5:**
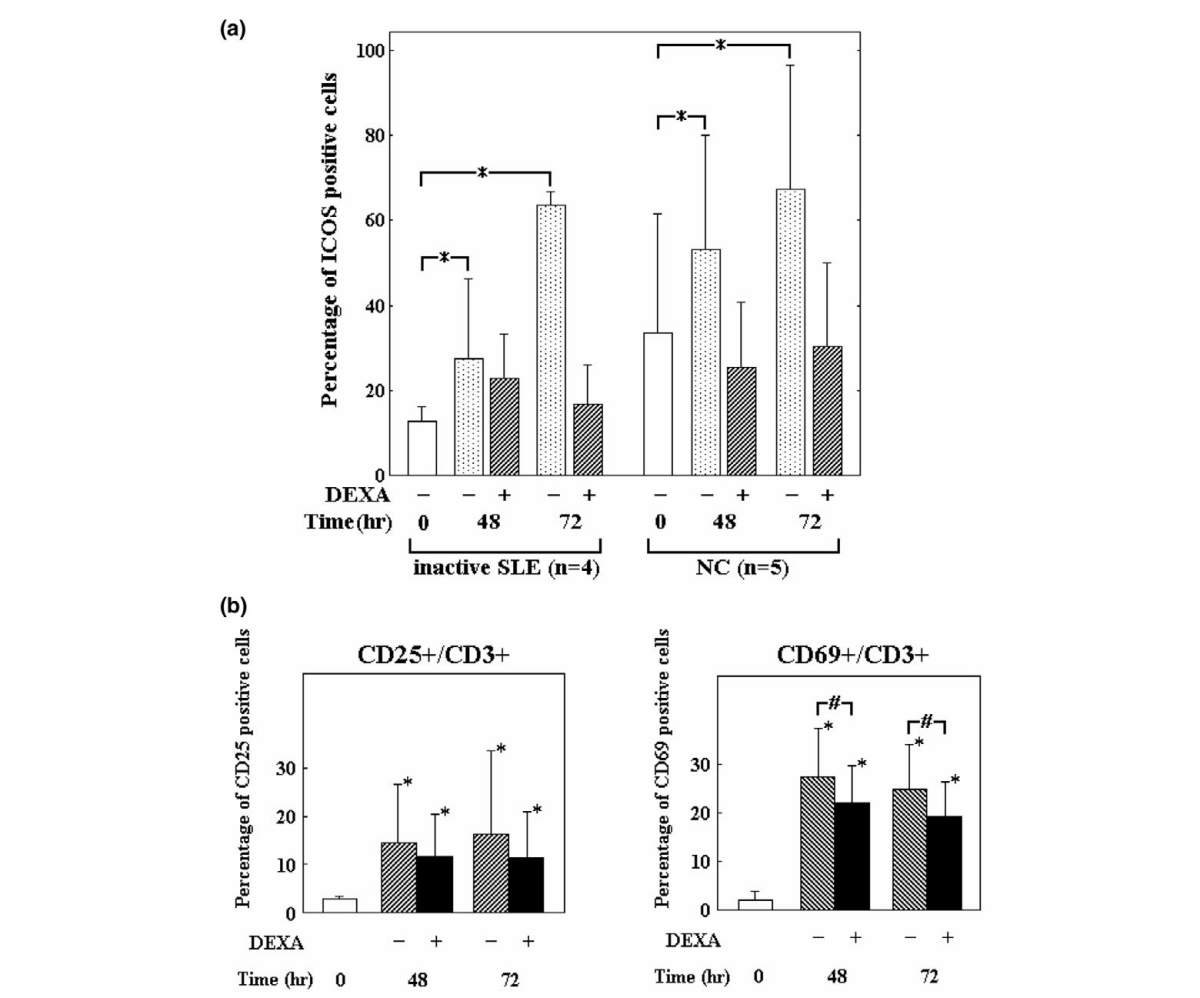
Effects of dexamethasone on ICOS expression after T cell activation. **(a) **Peripheral blood T cells from patients with inactive SLE (*n *= 4) and normal control individuals (*n *= 5) were cultured with ICOS co-stimulation for 48 or 72 hours in the presence or absence of 10^-6 ^mol/l dexamethasone and were analyzed using three-colour staining (anti-CD3-PerCP, anti-CD45RO-PE, biotinylated JTA009 plus streptavidin-FITC) and flow cytometry for ICOS expression. ICOS co-stimulation significantly induced ICOS expression on CD3^+^CD45RO^+ ^T cells in both patients with inactive SLE and normal control individuals (dotted columns). Dexamethasone at 10^-6 ^mol/l almost completely abrogated the induction of ICOS after ICOS co-stimulation (hatched columns). The Y-axis showes percentages of ICOS^+ ^cells among CD3^+^CD45RO^+ ^cells. **(b) **Normal peripheral blood T cells (*n *= 4) were cultured with ICOS co-stimulation for 48 or 72 hours in the presence or absence of 10^-6 ^mol/l dexamethasone and were analyzed using two-color staining (left panel, anti-CD3-FITC and anti-CD25-PE; right panel, anti-CD3-FITC and anti-CD69-PE) and flow cytometry. *P < 0.05 versus before stimulation, by Wilcoxon rank sum test. #*P *< 0.05 versus without dexamethasone, by Wilcoxon rank sum test. DEXA, dexamethasone; FITC, fluorescein isothiocyanate; ICOS, inducible co-stimulator; NC, normal control; PE, phycoerythrin; PerCP, peridinin chlorophyll protein; SLE, systemic lupus erythematosus.

We then examined effects of corticosteroid on induction of ICOS after ICOS co-stimulation of peripheral blood T cells. This is because all the patients except one with inactive SLE were receiving maintenance doses of corticosteroid whereas 13 out of the 16 patients with active SLE considered in the analysis of ICOS expression were examined before institution of any treatments and the remaining three patients with active disease were receiving 2.5, 15 and 30 mg/day prednisolone. In this experiment, we used dexamethasone (Sigma-Aldrich, St. Louis, MO, USA) instead of prednisolone. Dexamethasone at 10^-6 ^mol/l almost completely abrogated the induction of ICOS 72 hours after ICOS co-stimulation in both patients with inactive SLE and normal control individuals (Figure 5a). Results with dexamethasone at higher concentrations were essentially the same (data not shown). Inhibitory effects of dexamethasone on the induction of CD25 and CD69 with ICOS co-stimulation were less prominent (Figure 5b), indicating that ICOS is more sensitive to treatment with dexamethasone.

We also examined percentages of apoptotic cells with Annexin-V staining (Annexin V-FITC Apoptosis Detection Kit; BioVision, Mountain View, CA, USA). Treatment with dexamethasone at 10^-6 ^mol/l did not increase the percentages of Annexin-V positive T cells in gating of lymphocytes on flow cytometry 48 and 72 hours after ICOS co-stimulation (with and without dexamethasone, respectively: at 48 hours, 2.9 ± 1.0% and 1.7 ± 0.9%; at 72 hours, 0.7 ± 0.2% and 0.6 ± 0.3%). These data indicate that the relatively low expression of ICOS on peripheral blood T cells from patients with inactive SLE could be accounted for by treatment with maintenance doses of corticosteroid. These data also suggest that ICOS co-stimulation enhances the expression of ICOS on T cells and amplifies their response to ICOS co-stimulation in both patients with SLE and normal control individuals, and would (at least in part) explain the discrepancy between the relatively low expression of ICOS on peripheral blood T cells (Figure 2) and augmented response to ICOS co-stimulation in inactive SLE (Figures 3 and 4).

### ICOS co-stimulated peripheral blood T cells from patients with active SLE enhanced anti-dsDNA antibody production by autologous B cells

Finally, we investigated the involvement of ICOS in pathogenic autoantibody production in SLE. We purified peripheral blood T cells and B cells from eight patients with active SLE with high serum anti-dsDNA antibody levels and reconstituted them at a ratio of 1:1 ratio. The reconstituted cells were cultured for seven days in the presence or absence of stimulation with either anti-CD3 mAb plus JTA009 or anti-CD3 mAb plus JMAb23 (as described above, under Materials and method). Because ICOS and CD28 belong to the CD28 superfamily and both of them provide positive co-stimulatory signal to T cells, we also stimulated the reconstituted cells with anti-CD3 mAb (0.1 μg/ml) plus anti-CD28 mAb (2.0 μg/ml) for seven days. The supernatants were collected and the concentrations of IgG anti-dsDNA antibody and total IgG were measured using ELISA. To evaluate the effects of co-stimulatory signals on anti-dsDNA antibody or total IgG production, the results were expressed as a co-stimulatory index, which was calculated as follows: (IgG anti-dsDNA antibody or total IgG production with co-stimulation)/(the IgG anti-dsDNA antibody or total IgG production with anti-CD3 mAb plus JMAb23 stimulation).

**Figure 6:**
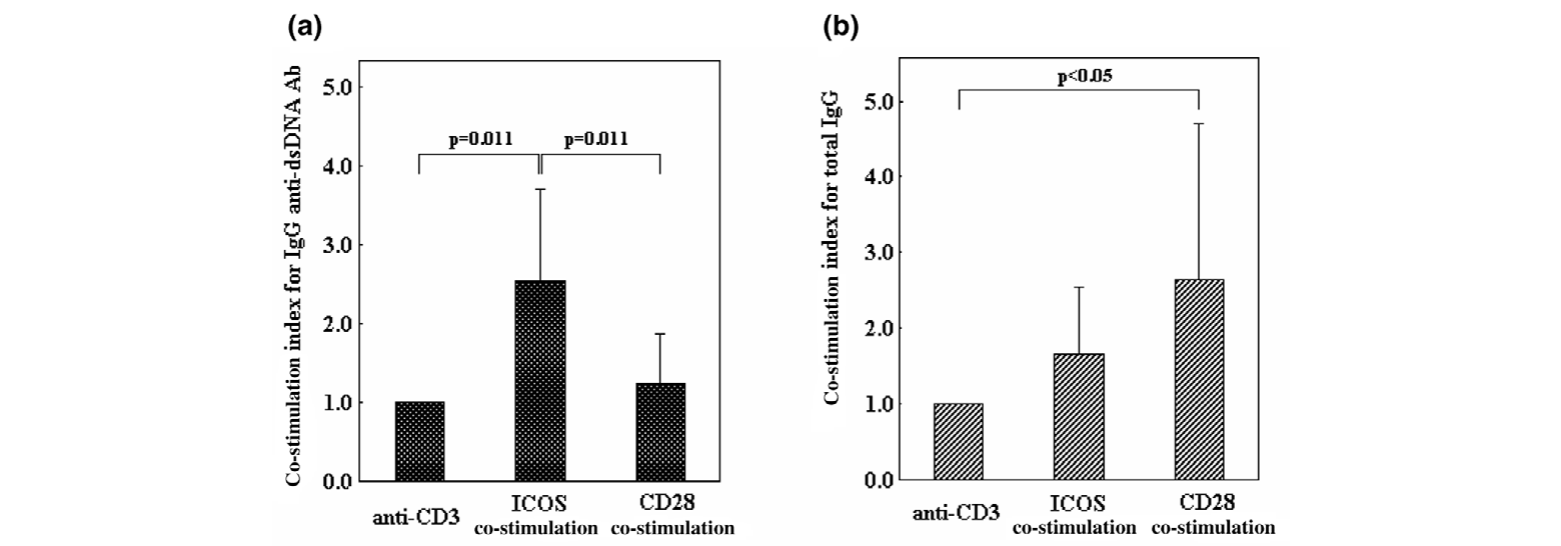
ICOS co-stimulated peripheral blood T cells in active SLE enhanced IgG anti-dsDNA antibody production by autologous B cells. Peripheral blood T cells and B cells were isolated from eight patients with active SLE, reconstituted at a 1:1 ratio and cultured in the presence of anti-CD3 mAb plus JTA009 (ICOS co-stimulation), anti-CD3 mAb plus anti-CD28 mAb (CD28 co-stimulation), anti-CD3 mAb plus JMAb23 (anti-CD3), or without stimulation for 7 days. IgG anti-dsDNA antibody and total IgG were determined by ELISA. The mean ± SD production of IgG anti-dsDNA antibody and total IgG, respectively, were as follows: anti-CD3 mAb plus JMAb23, 45.4 ± 64.4 U/ml and 274± 141 ng/ml; ICOS co-stimulation, 98.3 ± 118 U/ml and 475 ± 297 ng/ml; CD28 co-stimulation, 46.1 ± 64.1 U/ml and 734 ± 694 ng/ml; and without stimuli, 22.0 ± 29.7 U/ml and 216 ± 180 ng/ml. Co-stimulation indices for **(a) **IgG anti-dsDNA antibody and **(b) **total IgG were calculated as follows: the IgG anti-dsDNA antibody or total IgG production with co-stimulation/the IgG anti-dsDNA antibody or total IgG production with anti-CD3 mAb plus JMAb23. Differences between stimuli were evaluated using Wilcoxon rank sum test. Ab, antibody; ds, double stranded; ICOS, inducible co-stimulator; SD, standard deviation; SLE, systemic lupus erythematosus.

The co-stimulatory index for IgG anti-dsDNA antibody with ICOS co-stimulation was significantly higher than those with anti-CD3 mAb plus JMAb23 stimulation or CD28 co-stimulation. There was no significant difference between the latter two conditions (Figure 6a). Co-stimulatory index for total IgG production with CD28 co-stimulation, but not with ICOS co-stimulation, was significantly higher than that with anti-CD3 mAb plus JMAb23 stimulation (Figure 6b). These data indicate that ICOS co-stimulation selectively enhanced the production of IgG anti-dsDNA antibody in this reconstitution experiment. We also measured anti-tetanus antibodies in these culture supernatants by ELISA, but almost all the results were under the detection limit, except for some culture supernatants with large amounts of total IgG (data not shown).

To examine whether direct contact between T and B cells is required in the co-culture experiments, we separated T cells and B cells using filter inserts. Within one well, B cells were placed in the filter inserts whereas T cells were cultured under the filter inserts with or without the same stimuli as described above. In this culture system, T cells cannot stimulate B cells via surface molecules, but would be able to stimulate B cells via soluble factors secreted into the medium. The cells were cultured for seven days and the supernatants were collected. With or without stimulation, the separation of B cells from T cells using the filter inserts drastically decreased the production of IgG anti-dsDNA antibody by the co-cultures (data not shown). These data indicate that direct contact between T cells and B cells is required to augment the IgG anti-dsDNA antibody production of B cells by ICOS co-stimulated autologous T cells.

## Discussion

In the present study we investigated the expression and function of ICOS in SLE. The major findings of this study are as follows. First, JTA009 – a newly developed fully human anti-human ICOS mAb – specifically binds to ICOS with high avidity. Second, expression of ICOS was detected on a substantial proportion of peripheral blood T cells from normal control individuals. Third, expression of ICOS was augmented in peripheral blood CD4^+^CD45RO^+ ^T cells from patients with active SLE. Fourth, [^3^H]thymidine incorporation of peripheral blood T cells from patients with inactive SLE after ICOS co-stimulation was significantly higher than that for normal control individuals. Fifth, production of IFN-γ in the culture supernatant of peripheral blood T cells from patients with active and inactive SLE after ICOS co-stimulation was significantly increased compared with that in normal control individuals. Finally, induction of IgG anti-dsDNA antibody production by peripheral blood B cells by ICOS co-stimulated autologous T cells was relatively selective.

The expression of ICOS in resting T cells has been reported to be very low [9,32]. Sakamoto and coworkers [32] reported that 1.54%, 2.0% and 8.0% of peripheral blood T cells express ICOS in human, mouse and rat, respectively. In the present study, however, using the high-avidity anti-human ICOS mAb JTA009, we found that a substantial portion of human peripheral blood T cells do express ICOS. In both SLE patients and normal control individuals, ICOS was mainly expressed in CD45RO^+ ^T cells, which is consistent with the fact that CD45RO^+ ^T cells expressed ICOS more rapidly and strongly when they were stimulated with superantigens and human umbilical vein endothelial cells [43]. It has also been reported that the activation of T cells with CD28 co-stimulation or phorbol myristate acetate plus calcium ionophore strongly induces the expression of ICOS [10,12,32,44]. The significantly increased percentage of ICOS^+ ^cells and the significantly higher MFI with JTA009 in CD4^+^CD45RO^+ ^T cells from patients with active SLE therefore indicates that these T cells are already activated *in vivo *(Figure 2c,e). This possibility gains further support from the following results of the present study: expression of ICOS on peripheral blood T cells from patients with active SLE drastically decreased after treatment with high-dose prednisolone; ICOS co-stimulation significantly enhanced expression of ICOS on peripheral blood T cells from patients with inactive SLE and normal control individuals; and dexamethasone, a strong inhibitor of lymphocyte activation, almost completely abrogated the induction of ICOS with ICOS co-stimulation.

Recently, Hutloff and coworkers [45] also reported expression of ICOS and B7RP-1 in peripheral blood lymphocytes from patients with SLE using anti-ICOS mAb (F44) and anti-ICOSL mAb (HIL-131). The mean percentages of ICOS^+ ^cells for both CD4^+ ^and CD8^+ ^T cells using F44 were less than 5%, which were similar to the values obtained using SA12 but apparently lower than the values obtained using JTA009 (Table 1). Thus JTA009 did provide novel findings regarding the expression of ICOS on human peripheral blood T cells.

IFN-γ is a pivotal Th1 cytokine and has been involved in the immunopathogenesis of both murine and human lupus [34-40]. In mice, disruption of IFN-γ or IFN-γ receptor genes resulted in greatly reduced autoantibody production and organ destruction. Furthermore, treatment of MRL-Fas (lpr) mice with a plasmid encoding IFN-γ receptor-Fc fusion protein significantly ameliorated disease manifestations [46]. In the present study, we demonstrated that peripheral blood T cells from patients with active SLE spontaneously produced significantly larger amounts of IFN-γ and that ICOS co-stimulation induced significantly greater amounts of IFN-γ in peripheral blood T cells from both active and inactive SLE patients compared with normal control individuals (Figure 4a,b). We also observed significantly higher IFN-γ production by peripheral blood T cells from patients with inactive SLE with anti-CD3 mAb plus anti-CD28 mAb stimulation compared with normal control individuals. The excessive production of IFN-γ by peripheral blood T cells in response to ICOS as well as CD28 co-stimulation may be relevant to the immunopathogenesis of human SLE. ICOS co-stimulation also significantly increased the production of both IL-4 and IL-10 in peripheral blood T cells from the patients with SLE and normal control individuals, which were compatible with previous reports [42].

ICOS gene knockout mice are defective in germinal centre formation, antibody production and class switching in response to various antigens [13,47]. The ICOS-B7RP-1 interaction in mice is involved in the initial clonal expansion of primary and primed Th1 and Th2 cells in response to immunization and is important for its ability to support the B cell response [14]. Treatment of lupus model mice with anti-ICOS mAb resulted in reduced anti-dsDNA antibody in sera and renal pathology [22]. Recently, a novel RING-type ubiquitin ligase family member, Roquin, has been identified as an autoimmune regulator. Disrupted *roquin *in sanroque mice leads to over-expression of ICOS and IL-21 in T cells, unrestrained formation of follicular helper T cells, autoantibody production and lupus phenotype [48]. These data suggest the possibility that the ICOS-B7RP-1 interaction can also promote autoantibody production in human SLE. Indeed, ICOS co-stimulated T cells, but not CD28 co-stimulated T cells, from patients with active SLE supported IgG anti-dsDNA antibody production (Figure 6a). In contrast to IgG anti-dsDNA antibody production, total IgG production did not increase significantly by ICOS co-stimulation, which suggests the relative selectivity of the co-stimulation for IgG anti-dsDNA antibody production (Figure 6b).

## Conclusion

The data presented here indicate that ICOS co-stimulation is involved in the immunopathogenesis of SLE via the stimulation of proliferation of and cytokine production by T cells, and supporting IgG anti-dsDNA antibody production. Blockade of the ICOS-B7RP-1 interaction may be a candidate novel strategy for the treatment of this intractable autoimmune disease.

## Abbreviations

B7RP-1 = B7-related protein-1; ds = double stranded; ELISA = enzyme-linked immunosorbent assay; FITC = fluorescein isothiocyanate; ICOS = inducible costimulator; IFN = interferon; IL = interleukin; mAb = monoclonal antibody; KLH = keyhole limpet hemocyanin; MFI = mean fluorescence intensity; PBL = peripheral blood lymphocyte; PBS = phosphate-buffered saline; PE = phycoerythrin; PerCP = peridinin chlorophyll protein; SD = standard deviation; SLE = systemic lupus erythematosus; SLEDAI = Systemic Lupus Erythematosus Disease Activity Index; Th = T-helper (cell).

## Competing interests

Katsunari Tezuka is an employee of Japan Tobacco, Inc. All other authors declare that they have no competing interests.

## Authors' contributions

MK carried out fluorescence-activated cell sorting analysis and ELISA for anti-dsDNA antibody, and prepared the manuscript. M Harigai conceived the study and contributed to the preparation of the manuscript. M Hara contributed to the concept and interpretation of the study and separation of lymphocytes. Y Kawaguchi performed ELISA for human IgG. KT developed antibodies to human ICOS. MT and TS participated in ELISA for cytokines. Y Katsumata and SH carried out fluorescence-activated cell sorting analysis. CF and HI carried out proliferation assays. NK made contributions to the design and coordination of the study. All authors read and approved the final manuscript.

## Figures and Tables

**Table 1:** Characterization of JTA009

	JTA009 (%)	SA12 (%)	*P*
CD4^+^ICOS^+^/CD4^+^	29.2 ± 22.1	3.8 ± 2.4	0.0033
CD8^+^ICOS^+^/CD8^+^	11.6 ± 11.2	1.6 ± 1.0	0.0033
CD4^+^CD45RO^+^ICOS^+^/CD4^+^CD45RO^+^	37.3 ± 25.8	5.4 ± 4.0	0.0033
CD8^+^CD45RO^+^ICOS^+^/CD8^+^CD45RO^+^	17.1 ± 15.2	2.1 ± 1.5	0.0033

Peripheral blood T cells from 11 normal control individuals were multicolour stained and analyzed using flow cytometory. Values are expressed as mean ± SD in 11 normal control individuals. Wilcoxon rank sum test was used for the comparison of data between JTA009 and SA12.
